# A process-based dynamic model for succinic acid production by *Actinobacillus succinogenes*: regulatory role of ATP/ADP balance

**DOI:** 10.3389/fmicb.2025.1512982

**Published:** 2025-03-06

**Authors:** Emiliano Salucci, Fabrizio Cartenì, Francesco Giannino, Elisabetta de Alteriis, Francesca Raganati, Stefano Mazzoleni

**Affiliations:** ^1^Department of Agricultural Sciences, University of Naples “Federico II”, Portici, Italy; ^2^Faculty of Science and Engineering, Åbo Akademi University, Turku, Finland; ^3^Department of Biology, University of Naples “Federico II”, Napoli, Italy; ^4^Department of Chemical Engineering of Materials and Industrial Production, University of Naples “Federico II”, Napoli, Italy

**Keywords:** fermentation, bioreactors, system dynamics modeling, microbial growth models, energy balance

## Abstract

**Introduction:**

Succinic acid is an important chemical compound for biotechnological productions, being used as a basic platform to produce many industrial products in major business applications. It can be produced as fermentation end-product of anaerobic metabolism of different bacterial species, among which *Actinobacillus succinogenes* is largely used. Modeling microbial metabolic processes in controlled bioreactor systems is recognized as a useful tool to optimize growth conditions aimed at maximizing yield.

**Methods:**

A novel model is presented based on System Dynamics approach in which the maintenance of the ATP/ADP balance is introduced as a key regulatory process of *A. succinogenes* metabolism.

**Results and discussion:**

Model simulations accurately reproduce microbial growth and succinic acid production in anaerobic batch cultures at different initial glucose concentrations. Results reveal that the main limitations to maximal succinic acid production are glucose uptake restrictions and energy homeostasis costs (ATP/ADP balance) of the microbial population. The process-based modeling approach effectively describes the main metabolic processes and their regulation, providing a useful tool to define working conditions and overcome the criticalities of the SA fermentation process.

## 1 Introduction

Succinic acid (SA) is a C-4 dicarboxylic acid that has gained great interest because of its use as a precursor for several synthetic resins, chemical reagents, herbicides, fungicides, biodegradable polymers detergents and inks, among other products (Rigaki et al., 2020; Corona-González et al., 2008). Fermentative SA production has been accomplished by using several strains, among which the facultative anaerobes *Actinobacillus succinogenes, Basfia succiniproducens*, and *Mannheiimia succiniciproducens* are the most used. The potential for bio-SA market is in the replacement of existing petrol-based SA (Prabhu et al., 2020). Therefore, SA is considered as one of the chemical platforms for the future development at industrial scale, and one of the fastest growing markets (Ercole et al., 2021; Stylianou et al., 2020).

SA is a fermentation end-product of the anaerobic metabolism of *A. succinogenes*. Starting from a glucose-based substrate, *A. succinogenes* can split its metabolic flux into two branches: (i) C3 pathway leading to formate, acetate, ethanol, and lactate production, and (ii) C4 pathway for succinate production. Several studies reported that the cellular global demand for ATP and cofactors (NADH produced in the C3 route) controls the glycolytic flux (Almqvist et al., 2016; Bradfield and Nicol, 2014; Vemuri et al., 2002). In anaerobic systems, where glucose cannot be completely oxidized, the microorganisms accumulate metabolic intermediates to maintain the redox balance (Vemuri et al., 2002; McKinlay et al., 2007). The increase of NADH supports SA production, since SA is a highly reduced fermentation product (the production of 2 moles of SA requires 4 moles of NADH), with such glucose conversion to SA being maintained during fermentation, even if cell density achieves an early stationary phase (Corona-González et al., 2008). In some cases, the need of reducing equivalents is ensured by providing additional reduced carbohydrates (e.g., sorbitol) (Chatterjee et al., 2001) or increasing NADH availability (Sánchez et al., 2005; Zhu et al., 2014). Both growth and succinic acid production are limited by culture pH, which tends to decrease following acids accumulation (Corona-González et al., 2008). Another critical factor in the fermentation is the initial high glucose concentration which inhibits cell growth, an effect mainly attributed to osmotic stress causing cell damage (Pateraki et al., 2016b).

Several different models have been reported to describe the fermentative SA production by various microorganisms (wild type or genetically modified), from different carbon sources (in either pure forms or occurring in waste streams) (Song and Lee, 2006; Akhtar et al., 2014). In some cases, the substrate and product inhibition phenomena in the process have been considered in the models. Lin et al. (2008) reported an unstructured model that predicts SA production by *A. succinogenes* cultivated on glucose and wheat hydrolysates. The model succeeded in describing the inhibitory kinetics caused by both externally added chemicals and the same chemicals produced during fermentation. Corona-González et al. (2008) reported a kinetic study of glucose conversion to SA by *A. succinogenes*, where substrate and product inhibition phenomena were described by Jerusalimsky equations. Song et al. (2008) proposed a model for the SA production by *M. succiniciproducens* from glucose using a modified Monod model incorporating inhibition of both glucose and acids accumulated in the fermentation broth. Using another carbon source as glycerol, Li et al. (2010) presented the inhibitory effects by major products in the SA fermentation by *E. coli* mutants. They proposed a logistic model to describe the overall synergistic inhibitory effects. Vlysidis et al. (2011) reported a model of SA production by *A. succinogenes*, where a modified Monod equation considered both substrate and product inhibition. Pateraki et al. (2016a) developed unstructured models, including both substrate and product inhibition, that predicted the cultivation of *A. succinogenes* and *Basfia succiniciproducens* on a mixture of C5 and C6 sugars used to mime the sugar composition present in spent sulphite liquor. More recently, Rigaki et al. (2020) investigated the dissolved CO_2_ effect on SA production by *A. succinogenes* and proposed a double substrate model (glycerol and CO_2_) in batch reactors, which succeeded in effectively predicting the transient concentrations of glycerol and MgCO_3_.

In a more general context, a powerful modeling approach is based on System Dynamics tools (Forrester, 1961) allowing to develop process-based models of simplified metabolic pathways avoiding fully explicit fluxomic models' representations (de Falco et al., 2022), but still able to capture the main emergent dynamics of biomass and main metabolites production. Examples of applications of this type of modeling have been proposed to describe the main metabolic dynamics of the yeast *S. cerevisiae* (Mazzoleni et al., 2015), as well as of two bacterial species of biotechnological interest, such as *E. coli* and *B. subtilis* (Carteni et al., 2020). These works highlighted the relevance of self-produced growth inhibitors during cell proliferation that, in long runs of fed-batch cultures, may reduce the maximal achievable cell density. Very recently, de Alteriis et al. (2023) experimentally demonstrated in yeast fed-batch cultures that such inhibitory compound was self-DNA released by the live cells and accumulated in the culture medium.

Moreover, it is known that the production of target metabolic products can be either enhanced or worsened according to intracellular ATP content manipulation (Wisselink et al., 2007), so that ATP can be supplied to regulate the production (Zhou et al., 2009).

Considering the relevance of ATP availability in cell metabolism, it is noteworthy to recall another general effect associated to the cell energy balance. As discussed by de Alteriis et al. (2018), the ATP dynamics during the glycolytic process can lead to an energy crisis in the presence of excess of glucose. This may seem counter-intuitive, but it is explained by the different dynamics of glycolytic reactions, because the first irreversible glucose phosphorylation reaction may lead to a rapid ATP depletion when the glucose-6-phosphate accumulation is not sufficiently processed by the following ATP-forming reactions. This logically explained the occurrence of the metabolic shift between respiration and fermentation in Crabtree positive yeasts and other microbial species (De Deken, 1966; Wolfe, 2005; Paczia et al., 2012), as an avoidance of the conditions determining the ATP energy crisis at high sugar concentration (de Alteriis et al., 2018).

To the authors' knowledge, so far, no modeling studies of bacterial batch cultures have considered the role of the energy balance (in terms of ATP/ADP ratio) in regulating cell growth dynamics. Based on these considerations, in this work we propose a novel process-based kinetic model able to describe the dynamics of *A. succinogenes* DSM 22257 growth on glucose as substrate at five different concentrations [data from Ferone et al. (2017)], taking into account the crucial role of the ATP/ADP ratio in cell metabolism. Thus, the proposed System Dynamics model of *A. succinogenes* metabolism addressed the regulation of glucose uptake and its effect on growth, and the related energy demand for maintaining the intracellular pH, on the growth and fermentation performances in this biotechnologically important bacterial species.

## 2 Materials and methods

The present model was developed to describe the growth of *A. succinogenes* and the consequent production of acetic, formic and succinic acid in a batch reactor focusing on the fine-tuned energy balance of the bacterial cell.

**Figure 1:**
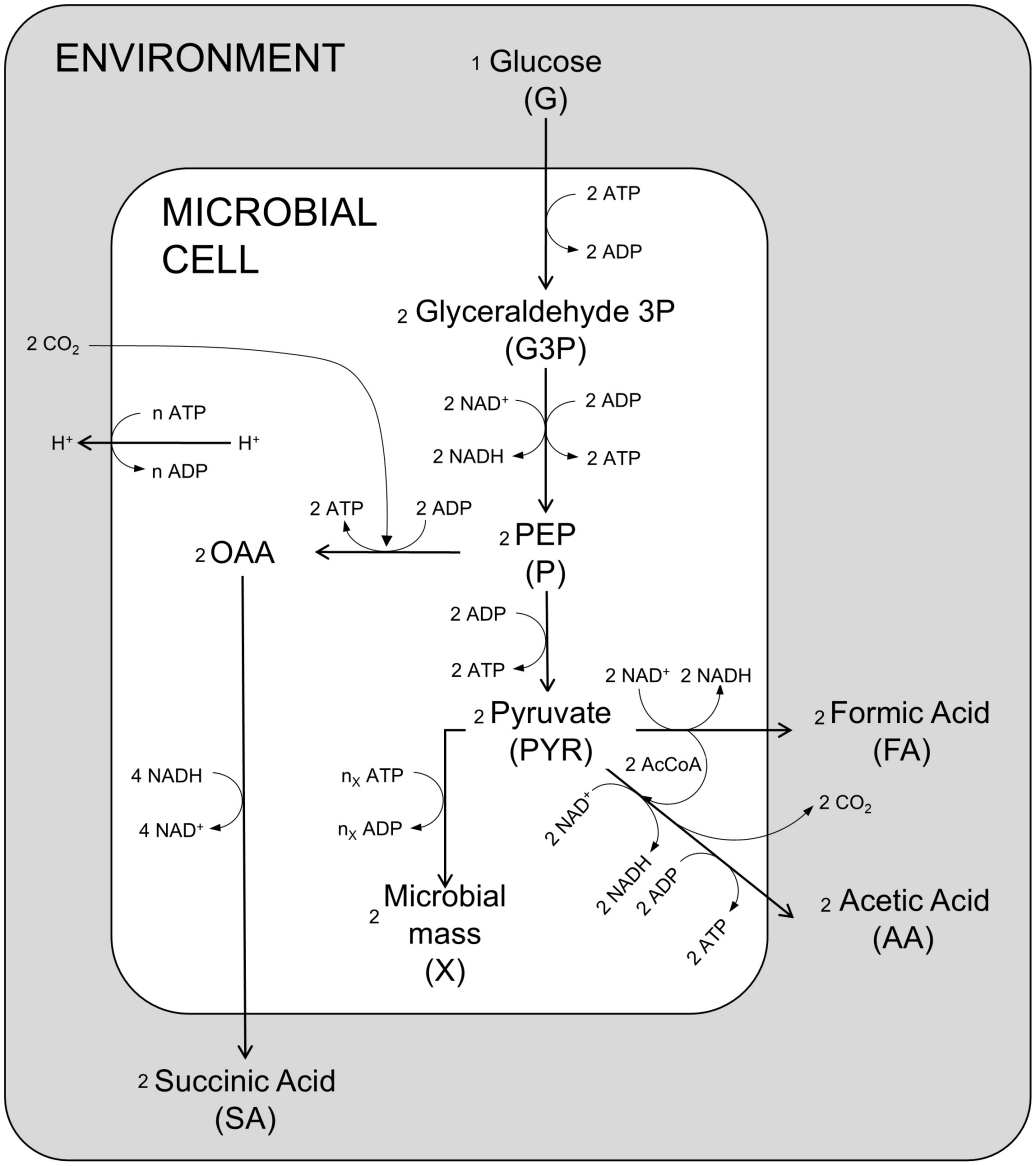
Schematic diagram of the main metabolic reactions of *A. succinogenes*. *OAA* oxaloacetate; *PEP* phosphoenolpyruvate; *AcCoA* acetyl coenzyme A.

Figure 1 represents the simplified metabolism of *A. succinogenes* in anaerobic conditions on glucose as carbon and energy source leading to the production of succinic, acetic and formic acids, as well as microbial mass, with the explicit representation of the main chemical reactions related to energy production and consumption involved in the processes. Although it is known that other by-products can be formed during glucose fermentation, including ethanol and lactic acid (C3 pathway), the specific strain used in Ferone et al. (2017) (*A. succinogenes* DSM 22257) does not produce lactic acid under the anaerobic experimental conditions, differently from other succinic producer species such as *Basfia succiniproducens* (Pateraki et al., 2016a). Regarding ethanol, it cannot be excluded that some ethanol may be produced by *A. succinogenes*, but in the experiments performed by Ferone et al. (2017), ethanol was not detected (pers. commun. by the authors).

**Figure 2:**
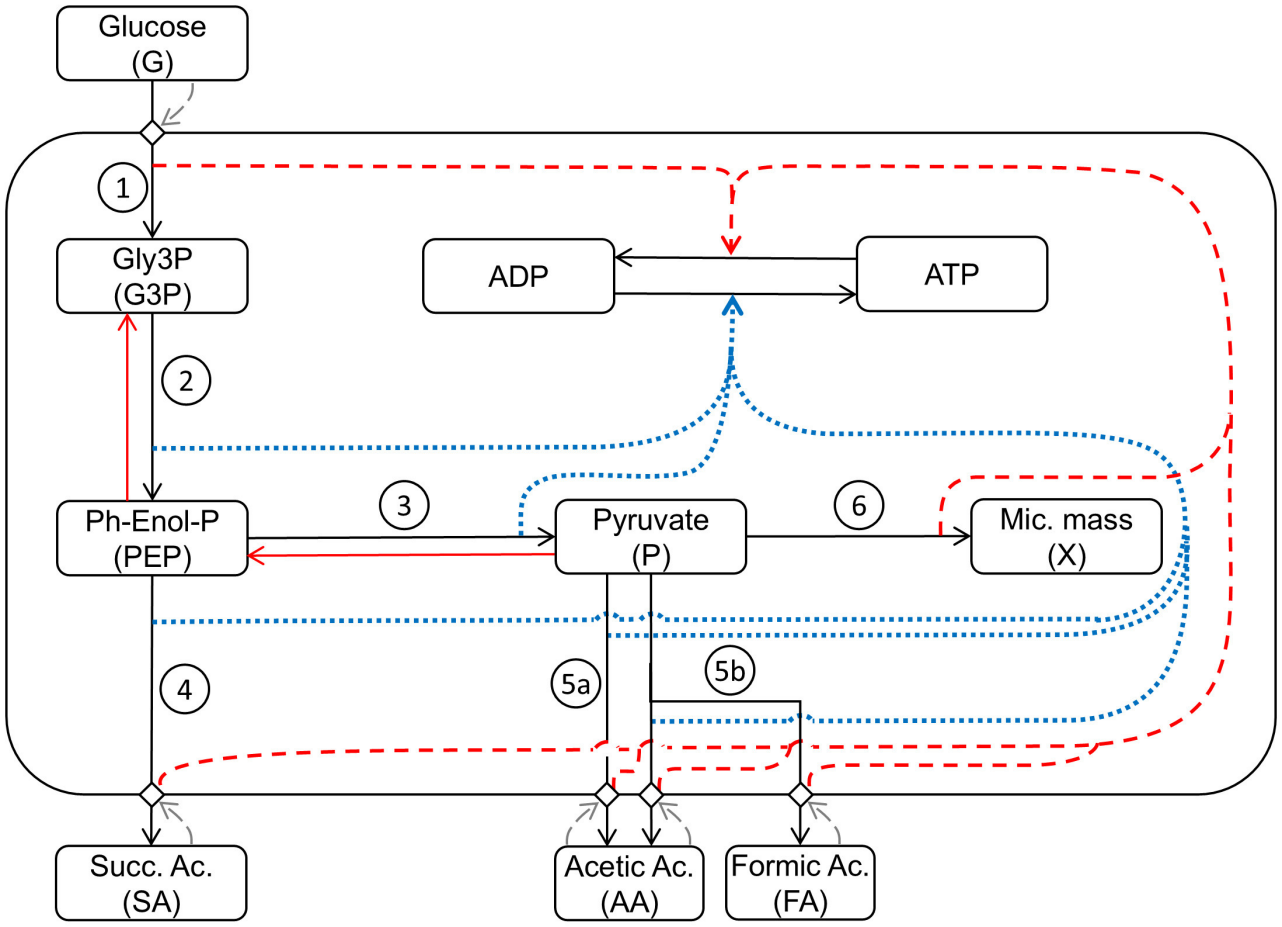
Schematic representation of the mathematical model structure and interactions between the considered metabolic pathways: (1) *Glycolysis*_1_: Glucose uptake/*G3P* production; (2) *Glycolysis*_2_: *PEP* production; (3) *Glycolysis*_3_: *P* production; (4) Production_1_: succinic acid production; (5a) *Production*_2_: acetic acid production; (5b) *Production*_3_: acetic and formic acid production; (6) *Growth*: microbial growth. *Black arrows* indicate direct metabolic reactions. *Red arrows* indicate reverse reactions. *Blue dotted arrows* indicate influence on ATP production. *Red dashed arrows* indicate influence on ATP consumption. *Grey dashed arrows* indicate response feedback effects.

Figure 2 shows a schematic diagram of the implemented processes in the model, providing a simplified representation of the complex network of the metabolism of *A. succinogenes* growing on glucose, producing the different acids, and where the influence of the different processes on the energy balance is taken into consideration affecting the ATP/ADP ratio inside the cell.

The state variables explicitly represented in the model are:

- the carbon source, *Glucose* (*G*) in the growth medium;
- the glycolysis intermediates: *Glyceraldehyde-3-phosphate* (*G3P*), *Phosphoenolpyruvate* (*PEP*) and *Pyruvate* (*P*);
- the fermentation products, i.e., *Succinic Acid* (*SA*), *Acetic Acid* (*AA*) and *Formic Acid* (*FA*);
- the microbial mass (*X*);
- the energy molecules, *ATP* and *ADP*.

The key assumption of the model is that the *A. succinogenes* metabolism level is controlled by the *ATP*/*ADP* ratio; in particular, this ratio is assumed to be regulated by growth energy costs and the related costs associated to the production of acids and their secretion. Indeed, the acids produced by fermentation are rapidly released into the reaction environment, but their extrusion from the cell is slowed down over time because they are displaced against a concentration gradient (Fukuzaki et al., 1990; Corona-González et al., 2008).

Further, in the model, specific response curves (*RC*) of the modeled processes to controlling variables are defined by logistic functions with two parameters, *k* which defines the slope of the curve and *mid* which represents the value of the controlling variable for which the RC is half of its maximum (middle). The slope of the logistic curve represents how fast the system responds when the threshold conditions are reached. All response curves, with exception of *RC*_*v*_, assume values between 0 and 1, slowing down the metabolic processes when specific threshold concentrations of the involved state variables are reached.

In the model, seven main metabolic processes and the energetic balance *ATP/ADP* are taken into account (Figure 2). The first modeled process is the *G* uptake by the cell population. This is followed by the glycolytic process, which produces *G3P* in the first energy investment phase. The chemical reactions condensed in this first step are spontaneous, irreversible, and consume *ATP*, representing a fundamental regulatory point for glycolysis (Mor et al., 2011). The second set of reactions represents the production of *PEP* and *P* and the associated *ATP* production. These reactions are reversible (Boiteux and Hess, 1981) and produce the substrates used in the different fermentation routes leading to *SA, AA*, and *FA*. The step for producing *SA* in the presence of CO_2_, sees *PEP* as a reagent and oxaloacetate as starting intermediate product, with consequent production of energy leading to the final conversion into *SA* (Figure 1). Starting from *P*, two different fermentative pathways are available: the first promotes the release of CO_2_ with consequent production of *ATP* and *AA*; the second route allows the formation of *FA* and subsequently, passes through the intermediate *AcetylCoA*, which produces *ATP* and *AA*. Due to the anaerobic conditions, *A. succinogenes* cannot re-assimilate the produced acids through the respiratory processes; so, they accumulate in the medium.

In the model (Figure 2), the role of pyruvate is essential being assumed as the main building block for the biosynthesis of microbial mass (Wang et al., 2019). As in previous modeling work (Mazzoleni et al., 2015; Carteni et al., 2020), the model also considers the accumulation *I* in the medium inhibiting all metabolic processes.

The uptake of *G* and conversion to *G3P* is formulated as a Michaelis–Menten (MM) kinetics which, being an irreversible process, depends only on the substrate *G* with a first order dependency on the cell mass (*X*) that exerts the process. The process is formulated as follows:


(1) $$\begin{array}{r} GLYCOLYSIS_{1}=v_{Gly1}\cdot RC_{v1}\cdot RC_{v2}\cdot \;\frac{G}{k_{Gly1}+G}\cdot \\ X\cdot \left(1-\frac{G3P}{{G3P}_{\max}}\cdot \frac{MW_{G3P}}{MW_{X}}\right)\cdot RC_{ATP} \end{array}$$

where *v*_*Gly*1_ is the maximum rate of the process, *k*_*Gly*1_ is the half-saturation constant and *MW*_*X*_ represents the molecular weight of *A. succinogenes* obtained from the composition of its biomass CH_2_O_0.5_N_0.18_ (Samuoelov et al., 1990). *MW*_*G*3*P*_ is the molecular weight of *G3P*, while *G3P*_*max*_, represents its maximum concentration within the cell (McKinlay et al., 2007). *RC*_*v*1_ represents the response curve function to the external concentration of *G*. In the case of limiting glucose concentration, the cell maximizes the glucose uptake rate, upregulating the phosphotransferase system (ptsG) whereas, at high *G* concentration, the uptake rate is reduced (Plumbridge, 1998; Neumann et al., 2012). This *G* uptake regulation is then formulated by two *RCs*, the first, describing the increased uptake rate at low glucose concentrations, formulated as:


(2) $$\begin{array}{l} RC_{v1}=1-\frac{a_{1}}{1+e^{\left(-k_{1}\cdot \left(G-\frac{G_{mid1}}{MW_{G}}\right)\right)}} \end{array}$$

where *a*_1_ and *k*_1_ are, respectively, the response curve affinity coefficient and the slope affinity, *MW*_*G*_ is the molecular weight of *G*, while *G*_*mid*1_ is the *G* middle concentration.

A second response curve, *RC*_*v*2_, affects the uptake rate, slowing down the process in cases of high glucose availability, as defined by the following formulation:


(3) $$\begin{array}{l} RC_{v2}=a_{2}+\frac{1-a_{2}}{1+e^{\left(-k_{2}\cdot \left(\log\left(\frac{G}{X}\right)-R_{mid}\right)\right)}} \end{array}$$

where *a*_2_ and *k*_2_ represent, respectively, the response curve affinity coefficient or minimum value to which the response curve tends, and the slope affinity, while *R*_*mid*_ is the glucose/microbial mass ratio middle concentration. In this case, the logistic curve used is defined as a function of the logarithm of the *G*/*X* ratio due to the different orders of magnitude of this ratio assumes in different conditions.

The processes requiring *ATP* or *ADP* present a specific *RC*_*ATP*_, which is a function of the ratio between the available chemical energy and the microbial mass *X* and it is formulated as:


(4) $$\begin{array}{l} RC_{ATP\;}=\frac{1}{1+e^{\left(-k_{3}\cdot \left(\frac{ATP}{X\cdot MW_{X}}-ATP_{mid}\right)\right)}} \end{array}$$

where *k*_3_ is the energy slope affinity, while *ATP*_*mid*_ is the *ATP* middle concentration. The *ADP* response curve (*RC*_*ADP*_) is equivalent to 1 minus the *RC*_*ATP*_.

The process leading to the production of *PEP* in the cell of *A. succinogenes*, formulated as the MM kinetics of a reversible process, is formulated as follows:


(5) $$\begin{array}{r} GLYCOLYSIS_{2}=v_{Gly2}\cdot \;\frac{G3P}{k_{Gly2}+G3P}\cdot X\\ \cdot \left(1-\frac{PEP}{PEP_{\max}}\cdot \frac{MW_{PEP}}{MW_{X}}\right)\cdot RC_{ADP} \end{array}$$

where *v*_*Gly*2_ is the maximum rate of this process, *k*_*Gly*2_ is the half-saturation constant, *MW*_*PEP*_ is the molecular weight of *PEP*, while *G3P* is the reaction substrate. As this is a reversible step in the glycolysis process, it is limited by the saturation of the product (*PEP*), therefore *PEP*_max_ represents its maximum concentration.

The glycolytic pathway ends with the production of *P* described by an MM kinetics in which the only substrate is *PEP*. The process is formulated as follows:


(6) $$\begin{array}{r} GLYCOLYSIS_{3}=v_{Gly3}\cdot \;\frac{PEP}{k_{Gly3}+PEP}\cdot X\\ \cdot \left(1-\frac{P}{P_{\max}}\cdot \frac{MW_{Pyr}}{MW_{X}}\right)\cdot RC_{ADP} \end{array}$$

where *v*_*Gly*3_ is the maximum rate of the process, *k*_*Gly*3_ is the half-saturation constant, *MW*_*P*_ is the molecular weight of *P*, while *P*_max_ represents the maximum concentration of the product, necessary in order to be able to define this process as reversible.

The processes that describe the different fermentation pathways always show MM kinetics, but change the substrate used in compliance with the reactive network. In particular, *Production*_1_ promotes the production of *SA* starting from *PEP, Production*_2_ allows to obtain only *AA* starting from *P* and finally, *Production*_3_ produces both *AA* and *FA* having *P* as substrate. These processes are defined in the following equations:


(7) $$\begin{array}{l} PRODUCTION_{1}=v_{P1}\cdot \frac{PEP}{k_{P1}+PEP}\cdot X\cdot RC_{ADP} \end{array}$$

(8) $$\begin{array}{l} PRODUCTION_{2}=v_{P2}\cdot \frac{P}{k_{P2}+P}\cdot X\cdot RC_{ADP} \end{array}$$

(9) $$\begin{array}{l} PRODUCTION_{3}=v_{P3}\cdot \frac{P}{k_{P3}+P}\cdot X\cdot RC_{ADP} \end{array}$$

where *v*_*P*1,2,3_ and *k*_*P*1,2,3_ are the maximum rate and the half-saturation constant, respectively, of the fermentative process considered.

Cell duplication in the bioreactor is described by *Growth* process which exhibits *P*-dependent MM kinetics as a substrate. The process is described by the following equation:


(10) $$\begin{array}{l} GROWTH=v_{G}\cdot \;\frac{P}{k_{G}+P}\cdot \;X\cdot RC_{ATP}\cdot RC_{G} \end{array}$$

where *v*_*G*_ and *k*_*G*_ are the maximum rate and the half-saturation constant, respectively. *RC*_*G*_ is the growth metabolic response curve which is a function of the external concentration of *G* and which inhibits it in conditions of lack of nutrients. The response curve is then formulated as:


(11) $$\begin{array}{l} RC_{G}=\frac{1}{1+e^{\left(-k_{4}\cdot \left(G-\frac{G_{mid2}}{MW_{G}}\right)\right)}} \end{array}$$

where *k*_4_ is the slope affinity, while *G*_*mid*2_ is the *G* middle concentration at which the phenomenon begins to become more relevant to the point of inhibiting the growth process.

Based on the above model description, the following eleven mass balance equations are defined:


(12) $$\begin{array}{l} \frac{dG}{dt}=-GLYCOLYSIS_{1} \end{array}$$

(13) $$\begin{array}{l} \frac{dG3P}{dt}=\frac{+\nu_{G3P}\cdot GLYCOLYSIS_{1}-GLYCOLYSIS_{2}}{X} \end{array}$$

(14) $$\begin{array}{l} \frac{dPEP}{dt}\\ =\frac{+\nu_{P}\cdot GLYCOLYSIS_{2}-GLYCOLYSIS_{3}-PRODUCTION_{1}}{X} \end{array}$$

(15) $$\begin{array}{l} \frac{dP}{dt}\\ =\frac{+\nu_{Pyr}\cdot GLYCOLYSIS_{3}-PRODUCTION_{2}-PRODUCTION_{3}-GROWTH}{X} \end{array}$$

(16) $$\begin{array}{l} \frac{dX}{dt}=\eta_{X}\cdot \nu_{X}\cdot GROWTH-SECRETION \end{array}$$

(17) $$\begin{array}{l} \frac{dSA}{dt}=+\nu_{SA}\cdot PRODUCTION_{1} \end{array}$$

(18) $$\begin{array}{l} \frac{dAA}{dt}=+\nu_{AA}\cdot \left(PRODUCTION_{2}+PRODUCTION_{3}\right) \end{array}$$

(19) $$\begin{array}{l} \frac{dFA}{dt}=+\nu_{FA}\cdot PRODUCTION_{3} \end{array}$$

(20) $$\begin{array}{l} \frac{dATP}{dt}=-\nu_{ATP1}\cdot GLYCOLYSIS_{1}+\nu_{ATP2}\cdot GLYCOLYSIS_{2}\\ +\nu_{ATP3}\cdot GLYCOLYSIS_{3}+\nu_{ATP4}\cdot \nu_{SA}\cdot PRODUCTION_{1}\\ +\nu_{ATP5}\cdot \nu_{AA}\cdot \left(PRODUCTION_{2}+PRODUCTION_{3}\right)\\ -\;\eta_{X}\cdot n_{X}\cdot \nu_{X}\cdot GROWTH-(n_{SA}\cdot \nu_{SA}\cdot PRODUCTION_{1}\\ \cdot RC_{SA}+n_{AA}\cdot \nu_{AA}\cdot (PRODUCTION_{2}+PRODUCTION_{3})\\ \cdot RC_{AA}+n_{FA}\cdot \nu_{FA}\cdot PRODUCTION_{3}\\ \cdot RC_{FA})+3\cdot \frac{ATP}{X}\cdot \frac{dX}{dt} \end{array}$$

(21) $$\begin{array}{l} \frac{dADP}{dt}=+\nu_{ATP1}\cdot GLYCOLYSIS_{1}-\nu_{ATP2}\cdot GLYCOLYSIS_{2}\\ -\nu_{ATP3}\cdot GLYCOLYSIS_{3}-\nu_{ATP4}\cdot \nu_{SA}\cdot PRODUCTION_{1}\\ -\nu_{ATP5}\cdot \nu_{AA}\cdot \left(PRODUCTION_{2}+PRODUCTION_{3}\right)\\ +\;\eta_{X}\cdot n_{X}\cdot \nu_{X}\cdot GROWTH+(n_{SA}\cdot \nu_{SA}\cdot PRODUCTION_{1}\\ \cdot RC_{SA}+n_{AA}\cdot \nu_{AA}\cdot (PRODUCTION_{2}+PRODUCTION_{3})\\ \cdot RC_{AA}+n_{FA}\cdot \nu_{FA}\cdot PRODUCTION_{3}\cdot RC_{FA})+3\cdot \frac{ADP}{X}\cdot \frac{dX}{dt} \end{array}$$

The state variables described in the mass balances are distinguished based on their location in the reaction environment. The microbial mass (*X*) and the extracellular chemical species (*G, SA, AA*, and *FA*) are expressed in moles while the intracellular chemical species (*G3P, PEP*, and *P*) are expressed in mol X^−1^. As for *ATP* and *ADP*, they are considered dimensionless due to their very low cell concentrations compared to all the other state variables (McKinlay et al., 2007).

The ν_*i*_ and *n*_*i*_ coefficients, present in all mass balances, represent the different stoichiometric ratios of the chemical reactions considered for each process.

Furthermore, the energy cost of the production and secretion of acids is assumed to increase with the external concentration of such acids. This phenomenon was modeled with three metabolic response curves which are a function of the external concentrations of *SA, AA*, and *FA* and which dynamically simulate the increase in resistance to extrusion (concentration gradient) of the fermentation products, formulated as:


(22) $$\begin{array}{l} RC_{SA}=\frac{1}{1+e^{\left({-k}_{5}\cdot \left(SA-\frac{SA_{mid}}{MW_{SA}}\right)\right)}} \end{array}$$

(23) $$\begin{array}{l} RC_{AA}=\frac{1}{1+e^{\left(-k_{6}\cdot \left(AA-\frac{AA_{mid}}{MW_{AA}}\right)\right)}} \end{array}$$

(24) $$\begin{array}{l} RC_{FA}=\frac{1}{1+e^{\left(-k_{7}\cdot \left(FA-\frac{FA_{mid}}{MW_{FA}}\right)\right)}} \end{array}$$

where *k*_5_, *k*_6_ and *k*_7_ are the slope affinity for each response curve; *SA*_*mid*_, *AA*_*mid*_ and *FA*_*mid*_ are the acid middle concentrations at which the phenomenon begins to be strongly inhibited; while *MW*_*SA*_, *MW*_*AA*_ and *MW*_*FA*_ are the molecular weight of the three acids.

As for the *ATP* and *ADP* balances, it was necessary to add a term that prevents the dilution over time of their cellular concentration due to microbial growth $\left(\left(\frac{ATP}{X}\cdot \frac{dX}{dt}\right)\;or\;\left(\frac{ADP}{X}\cdot \frac{dX}{dt}\right)\;\right)$.

The model has been used to simulate the anaerobic batch culture of *A. succinogenes* growing on glucose as carbon source of the experiments reported by Ferone et al. (2017). Cell death was not considered because it was not experienced in the experimental tests (Ferone et al., 2017).

The set of initial values of the state variables for each experiment (G1, G2, G3, G4, and G5) is described in Table 1. The initial values of *ATP* and *ADP* were obtained assuming that the sum of their concentrations in the cell is approximately constant. Their initial dimensionless quantity has been obtained by using Equation 20 by making two assumptions: (i) their sum (*s*_*ATP*_) = 1 mol X^−1^; (ii) their concentrations are perfectly equal at time *t*_0_:


(25) $$\begin{array}{l} ATP_{0}=\frac{s_{ATP}\cdot X\cdot MW_{X}}{2} \end{array}$$

**Table 1:** State variables initial values and simulation setup parameters.

**Symbol**	**Description**	**Unit**	**G1**	**G2**	**G3**	**G4**	**G5**
*G*_0_	Glucose initial value	*molG*	0.03	0.12	0.24	0.37	0.45
*G*3*P*_0_	Glyceraldehyde 3phosphate initial value	*molG*3*P molX*^−1^	0	0	0	0	0
*PEP*_0_	Phosphoenolpyruvate initial value	*molP molX* ^−1^	0	0	0	0	0
*P*_0_	Pyruvate initial value	*molPyr molX* ^−1^	0	0	0	0	0
*X*_0_	Microbial mass initial value	*molX*	1.05 × 10^−3^	1.93 × 10^−3^	7.25 × 10^−4^	1.07 × 10^−3^	5.64 × 10^−4^
*SA*_0_	Succinic acid initial value	*molSA*	0	0	0	0	0
*FA*_0_	Acetic acid initial value	*molAA*	0	0	0	0	0
*AA*_0_	Formic acid initial value	*molFA*	0	0	0	0	0
*ATP*_0_	Adenosine triphosphate initial value	−	1.29 × 10^−2^	2.37 × 10^−2^	8.90 × 10^−3^	1.31 × 10^−2^	6.92 × 10^−3^
*ADP*_0_	Adenosine diphosphate initial value	−	1.29 × 10^−2^	2.37 × 10^−2^	8.90 × 10^−3^	1.31 × 10^−2^	6.92 × 10^−3^
*t*_*END*_	Time of simulation end	*h*	60	100	100	100	100

Fixed and calibrated parameters are described in Tables 2, 3, respectively.

**Table 2:** Model's fixed parameters.

**Symbol**	**Description**	**Unit**	**Value**
*MW*_*X*_	Molecular weight *A. succinogenes*	*gX molX* ^−1^	24.55
*MW*_*G*_	Molecular weight glucose	*gG molG* ^−1^	180.16
*MW*_*G*3*P*_	Molecular weight glyceraldehyde 3-phosphate	*gG*3*P molG*3*P*^−1^	170.06
*MW*_*PEP*_	Molecular weight phosphoenolpyruvate	*gP molP* ^−1^	168.04
*MW*_*P*_	Molecular weight pyruvate	*gPyr molPyr* ^−1^	88.06
*MW*_*SA*_	Molecular weight succinic acid	*gSA molSA* ^−1^	118.09
*MW*_*AA*_	Molecular weight acetic acid	*gAA molAA* ^−1^	60.05
*MW*_*FA*_	Molecular weight formic acid	*gFA molFA* ^−1^	46.03
*s*_*ATP*_	*ATP* in microbial mass	*gX* ^−1^	1.00
ν_*G*3*P*_	Stoichiometric ratio *G3P*/*G*	*molG*3*P molG*^−1^	2.00
ν_*P*_	Stoichiometric ratio *P*/*G3P*	*molP molG*3*P*^−1^	1.00
ν_*Pyr*_	Stoichiometric ratio *Pyr*/*P*	*molPyr molP* ^−1^	1.00
ν_*X*_	Stoichiometric ratio *X*/*Pyr*	*molX molPyr* ^−1^	3.00
ν_*SA*_	Stoichiometric ratio *SA*/*P*	*molSA molP* ^−1^	1.00
ν_*AA*_	Stoichiometric ratio *AA*/*Pyr*	*molAA molPyr* ^−1^	1.00
ν_*FA*_	Stoichiometric ratio *FA*/*Pyr*	*molFA molPyr* ^−1^	1.00
ν_*ATP*1_	Stoichiometric ratio *ATP*/*G3P*	*molG* ^−1^	2.00
ν_*ATP*2_	Stoichiometric ratio *ATP*/*P*	*molG*3*P*^−1^	1.00
ν_*ATP*3_	Stoichiometric ratio ATP/*Pyr*	*molP* ^−1^	1.00
ν_*ATP*4_	Stoichiometric ratio *ATP*/*SA*	*molSA* ^−1^	1.00
ν_*ATP*5_	Stoichiometric ratio *ATP/AA*	*molAA* ^−1^	1.00

**Table 3:** Model calibrated parameters.

**Symbol**	**Description**	**Unit**	Initial value^*^	**Value**
*v*_*Gly*1_	Maximum glycolysis rate 1	*molG molX*^−1^ *h*^−1^	1.50	2.32
*k*_*Gly*1_	Glycolysis saturation constant 1	*molG*	1.00	39.99
*v*_*Gly*2_	Maximum glycolysis rate 2	*molG*3*P molX*^−1^ *h*^−1^	1.00	1.78
*k*_*Gly*2_	Glycolysis saturation constant 2	*molG*3*P molX*^−1^	1.00 × 10^−5^	1.24 × 10^−10^
*v*_*Gly*3_	Maximum glycolysis rate 3	*molP molX*^−1^ *h*^−1^	1.00	1.08
*k*_*Gly*3_	Glycolysis saturation constant 3	*molP molX* ^−1^	1.00 × 10^−5^	9.90 × 10^−5^
*v*_*P*1_	Maximum production rate 1	*molP molX*^−1^ *h*^−1^	1.00	0.27
*k*_*P*1_	Production saturation constant 1	*molP molX* ^−1^	1.00 × 10^−5^	2.65 × 10^−5^
*v*_*P*2_	Maximum production rate 2	*molPyr molX*^−1^ *h*^−1^	1.00	0.12
*k*_*P*2_	Production saturation constant 2	*molPyr molX* ^−1^	1.00 × 10^−5^	6.71 × 10^−4^
*v*_*P*3_	Maximum production rate 3	*molPyr molX*^−1^ *h*^−1^	1.00	0.20
*k*_*P*3_	Production saturation constant 3	*molPyr molX* ^−1^	1.00 × 10^−5^	3.70 × 10^−4^
*v*_*G*_	Maximum growth rate	*molPyr molX*^−1^ *h*^−1^	0.50	8.61 × 10^−2^
*k*_*G*_	Growth saturation constant	*molPyr molX* ^−1^	1.00 × 10^−5^	1.89 × 10^−4^
*G*3*P*_max_	Glyceraldehyde 3-phosphate max	*gG*3*P gX*^−1^	5.00 × 10^−4^	4.97 × 10^−4^
*PEP*_max_	Phosphoenolpyruvate max	*gP gX* ^−1^	5.00 × 10^−4^	4.70 × 10^−4^
*P*_max_	Pyruvate max	*gPyr gX* ^−1^	5.00 × 10^−4^	4.54 × 10^−4^
η_*X*_	Microbial mass efficiency	−	0.70	0.83
*n*_*X*_	Stoichiometric coefficient microbial mass	*molX* ^−1^	1.00	1.57
*n*_*SA*_	Stoichiometric coefficient succinic acid	*molSA* ^−1^	1.20	1.49
*n*_*AA*_	Stoichiometric coefficient acetic acid	*molAA* ^−1^	5.00	6.15
*n*_*FA*_	Stoichiometric coefficient formic acid	*molFA* ^−1^	2.50	3.01
*a*_1_	Response curve affinity coefficient 1	−	1.00	0.88
*k*_1_	Response curve slope affinity 1	*molG* ^−1^	2.00 × 10^+2^	53.39
*G*_*mid*1_	Glucose mid concentration 1	*gG*	5.00	51.49
*a*_2_	Response curve affinity coefficient 2	−	1.00	0.18
*k*_2_	Response curve slope affinity 2	−	2.00 × 10^+2^	10.13
*R*_*mid*_	Ratio G/X mid concentration	−	5.00	1.20
*k*_3_	Response curve slope *ATP*	*gX*	15.00	22.06
*ATP*_*mid*_	*ATP* mid concentration	*gX* ^−1^	0.25	0.36
*k*_4_	Response curve slope glucose	*molG* ^−1^	3.00 × 10^+2^	4.10 × 10^+2^
*G*_*mid*2_	Glucose mid concentration 2	*gG*	2.50	2.02
*k*_5_	Response curve slope succinic acid	*molSA* ^−1^	13.93	13.84
*SA*_*mid*_	Succinic acid mid concentration	*gSA*	20.00	23.05
*k*_6_	Response curve slope acetic acid	*molAA* ^−1^	5.00	11.04
*AA*_*mid*_	Acetic acid mid concentration	*gAA*	2.00	0.45
*k*_7_	Response curve slope formic acid	*molFA* ^−1^	5.00	8.67
*FA*_*mid*_	Formic acid mid concentration	*gFA*	1.00	1.08

* *Initial values of the calibrated parameters* *G*3*P*_max_, *PEP*_max_ *and* *P*_max_ *were estimated from McKinlay et al. (**2007**), whereas* *n*_*SA*_, *n*_*AA*_ *and* *n*_*FA*_ *from Li et al. (**2010**)*.

The mathematical equations were integrated using MATLAB R2023a (the MathWorks) with a variable order solver (ode15s). The model calibration was performed by minimizing the sum of the squared errors (SSE):


(26) $$\begin{array}{l} SSE=\overset{T}{\underset{k=1}{\sum }}\overset{S}{\underset{j=1}{\sum }}\overset{N}{\underset{i=1}{\sum }}\frac{{\left(C_{jdata,i}-C_{jsim,i}\cdot MW_{C}\right)}^{2}}{C_{jdata,\max}} \end{array}$$

where *C* represents the generic state variable for which experimental data are available (*G, X, SA, AA* and *FA*), *N* is the number of outputs available per experiment, *S* is the number of state variables, finally *T* represents the total number of tests conducted. Minimization was performed using the MATLAB *fminsearch* routine, which implements a Nelder-Mead simplex algorithm (Lagarias et al., 1998).

A sensitivity analysis was also implemented to observe the behavior of the model when subjected to parametric perturbation. Using a local sensitivity analysis (Morris, 1991; Norton, 2015), the sensitivity index was calculated by varying, one at a time, each calibrated parameter by ± 5%:


(27) $$\begin{array}{l} SSE_{m,\Delta }\\ =\sqrt{\frac{1}{n}\overset{2}{\underset{j=1}{\sum }}\overset{N}{\underset{i=1}{\sum }}\frac{{\left({C_{j}\left(p_{1},\ldots ,p_{m}+\Delta ,\ldots ,p_{n}\right)}_{.i}-{C_{j}\left(p_{1},\ldots ,p_{n}\right)}_{.i}\right)}^{2}}{{C_{j}\left(p_{1},\ldots ,p_{n}\right)}_{,\max}-{C_{j}\left(p_{1},\ldots ,p_{n}\right)}_{,\min}}} \end{array}$$

*SSEm*, Δ, is the standardized elemental effect of the p_m_ parameter, calculated with respect to biomass and succinic acid, perturbed by (± 5%), and calculated with respect to the value assumed by the state variable using the set of calibration parameters. Finally, *n* represents the number of parameters to be analyzed.

## 3 Results

**Figure 3:**
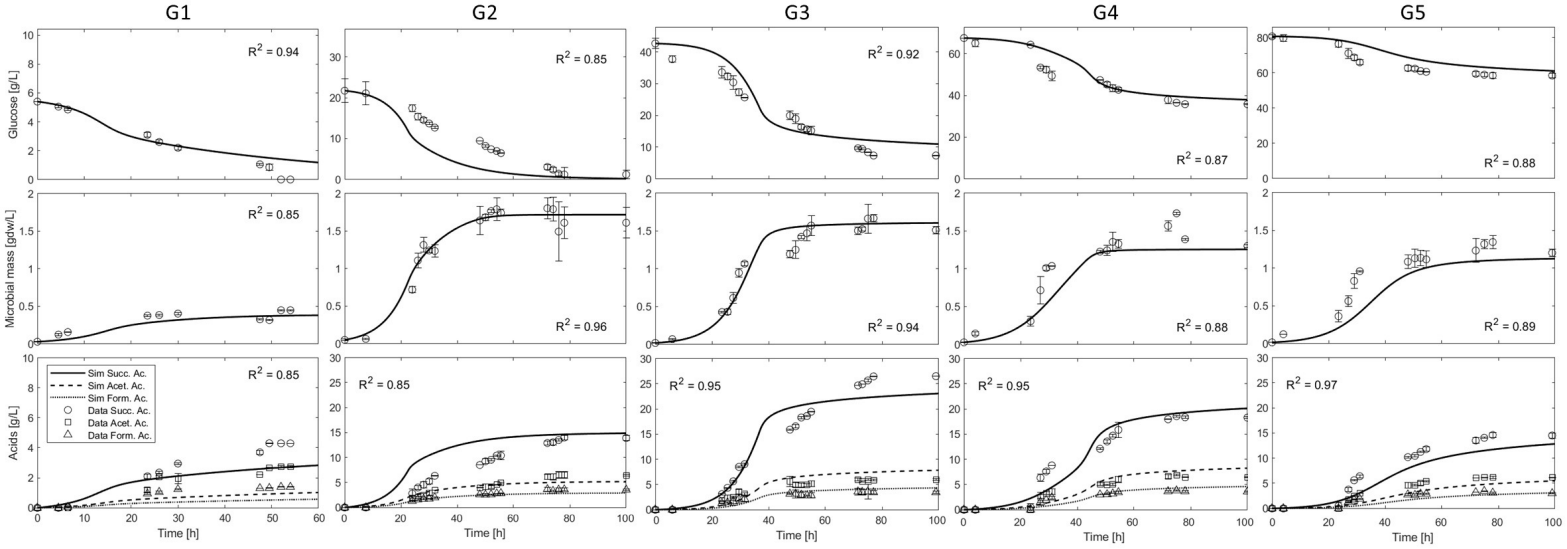
Simulations and experimental data of *A. succinogenes* growth and production of five batch experiments with different initial glucose concentrations: G1 = 5.40 g L^−1^; G2 = 21.75 g L^−1^; G3 = 42.65 g L^−1^; G4 = 67.45 g L^−1^; G5 = 80.70 g L^−1^ (Ferone et al., 2017).

The model has been used to simulate the anaerobic batch culture of *A. succinogenes* DSM 22257 strain growing on glucose as carbon source, as reported in batch fermentation tests performed at five different initial glucose concentrations, 5.40, 21.75, 42.65, 67.45, and 80.70 g L^−1^ (from here we will refer to them as G1, G2, G3, G4, and G5) (Ferone et al., 2017). The cells were cultivated in Pyrex anaerobic bottles (100 mL) containing 75 mL medium, under a nitrogen atmosphere. The fermentation medium per liter included: 5 g of yeast extract, 1 g of NaCl, 0.3 g of Na_2_HPO4, 1.4 g of NaH_2_PO4, 1.5 g of K_2_HPO4, 0.2 g of MgCl_2_·6H_2_O, and 0.23 g of CaCl_2_·2H_2_O. To serve as an indirect CO_2_ source and help maintain pH stability during cell growth, MgCO3 was added in suspension at an initial concentration of 5–30 g/L. All the fermentation tests were stopped when the concentrations of cells, glucose, and metabolites were constant for more than 24 h (Ferone et al., 2017). Figure 3 shows the model simulations and the corresponding experimental data of residual glucose, microbial mass, and acids in the medium, monitored during the entire time course of the five batch tests performed.

The observed trends in Figure 3 show that no significant initial lag phase under the reported conditions, i.e., the exponential cell growth phase was coupled with fast glucose consumption and simultaneous acid (*SA, AA*, and *FA*) production in all cases. The maximum cell density (about 1.60 g/L) was obtained in the experiment G2; higher concentrations of initial glucose (G3-5) resulted in the production of less biomass, clearly indicating an inhibition phenomenon. In all experiments, after about 30–40 h cell growth stopped, with the glucose consumption rate slowing down, while *FA* and *AA* concentrations approached a constant value. Instead, the principal fermentation product *SA* was continuously produced up to a final constant concentration, with an apparent decoupling between growth and its production. The maximum observed *SA* concentration was about 30.0 g/L in the experiment G3. At higher concentrations of glucose (G4 and G5), the maximum *SA* concentration decreased to values below 20.0 g/L. As regards the formation of the other two acids (*AA* and *FA*), the maximum concentrations remained fairly constant between the different experiments with the exception of G1. Comparatively, the concentrations of these acids (*AA* and *FA*) were significantly lower than that of SA, whatever the initial glucose concentration.

An interesting observation is that the microorganism consumed all the available sugar up to the concentration of ≈20 g/L (G1 and G2), whereas it was not able to consume all the supplied glucose in the case of higher initial sugar concentrations (G3, G4, and G5).

As can be seen from the simulation curves calibrated over the entire experimental dataset (Figure 3), the microbial mass, glucose and acids dynamics are well described, both in the exponential growth and in the slowdown phases. As can also be observed in Figure 3 (G1), although the microbial growth is well described, the mathematical model does not fully capture the acid production for this case, which is apparently underestimated. This result is due to the lower glucose uptake, which leads to incomplete glucose consumption, resulting in a correct final mass balance. On the other hand, Figure 3 (G2) clearly shows that the transient data for glucose consumption and succinic acid production are overestimated. Nevertheless, the model successfully describes the steady state of the bioreactor and its achievement in the last 20 h.

**Figure 4:**
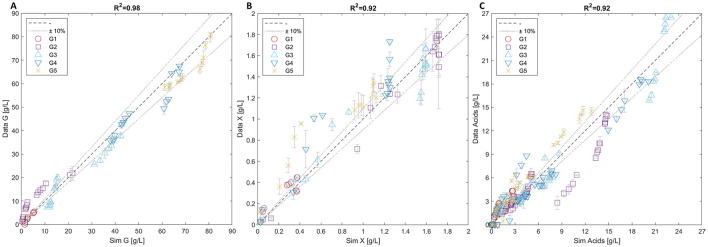
Parity plots of glucose **(A)**, microbial mass **(B)**, and acids **(C)**. Each plot includes data from all five batch experiments (G1-5).

Figure 4 provides a summary of the simulation results with the three parity plots showing a very good agreement between the measured and simulated values for all the experiments related to glucose, microbial mass and acids (Figures 4A–C, respectively).

**Figure 5:**
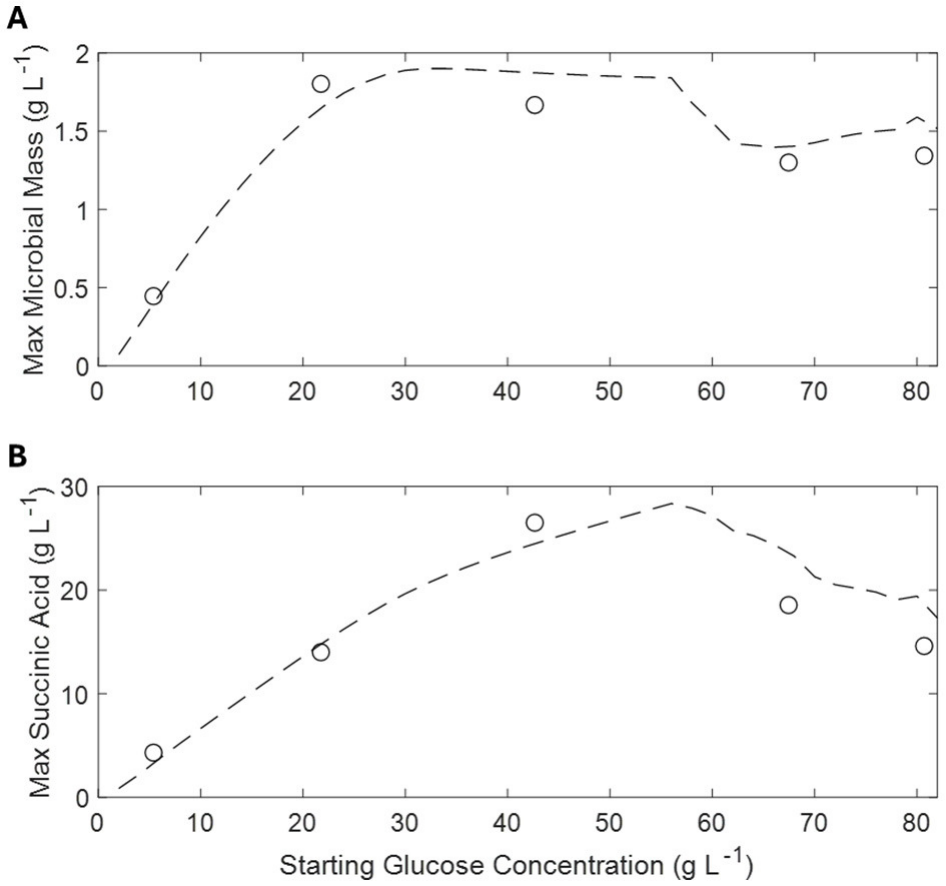
Simulated and observed maximum microbial mass **(A)** and corresponding succinic acid production at different glucose concentrations **(B)**. Simulations are indicated by *dashed lines* and observations by *open circles*.

Figure 5 shows the results, in terms of maximum achieved microbial mass and SA production, of repeated simulations of the batch cultures with increasing initial glucose concentration. Regarding the achieved microbial mass, the absolute maximum is achieved between 20 and 30 g L^−1^ of initial glucose then slightly decreasing at higher concentrations. On the other hand, SA production increases more slowly, peaking between 50 and 60 g L^−1^ of initial glucose while significantly decreasing at higher values.

**Figure 6:**
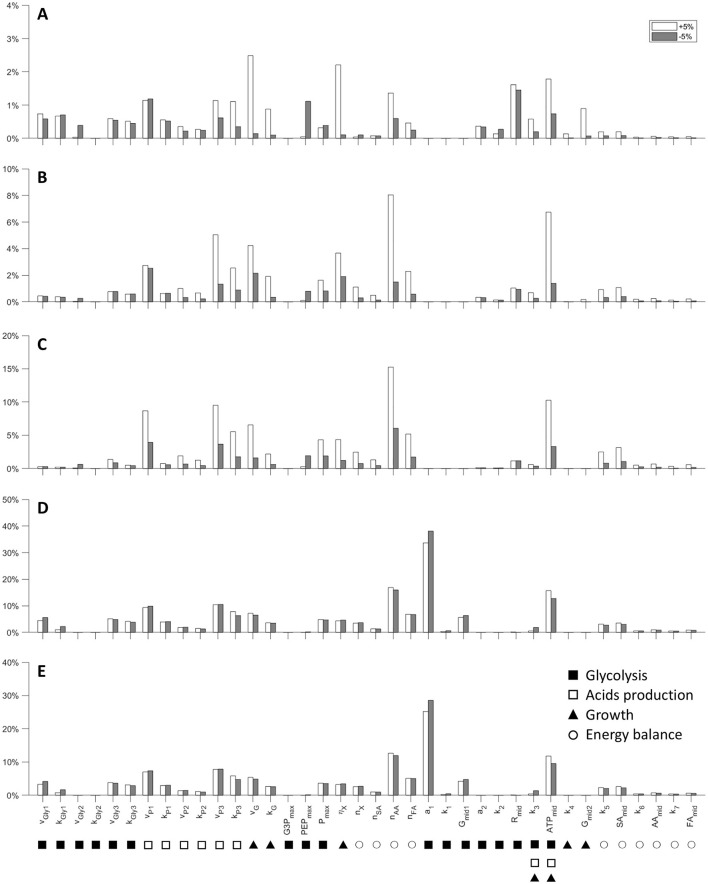
Model parameters local sensitivity analysis. Variation of the model outcome for changes (±5%) in the values of each parameter for all the experiment (see Material and methods for details). **(A)** G1 = 5.40 g L^−1^; **(B)** G2 = 21.75 g L^−1^; **(C)** G3 = 42.65 g L^−1^; **(D)** G4 = 67.45 g L^−1^; **(E)** G5 = 80.70 g L^−1^. Parameters are grouped in relation to the main processes they influence (symbols under each parameter name).

The results of the sensitivity analysis are presented in Figure 6. This purely mathematical analysis illustrates the responsiveness of the model to the variability of the parameters (±5%) in the evaluation of the state variables, using the estimated coefficients of the fit as a reference.

It is interesting to note that the model has the lowest sensitivity to parameters when simulating low initial concentration of glucose (G1) compared to other experiments (from G2 to G5). For instance, the mathematical analysis of case G1, in which both the acids produced, and the glucose consumed are underestimated, shows that the microbial growth parameters (*v*_*G*_, η_*G*_) have the greatest influence, with a total variation between 2% and 2.5%. The parameters related to glucose uptake (*v*_*Gly*1_, *k*_*Gly*1_, R_mid_) are the second most significant, which shows that even a 5 % increase in these parameters can lead to higher sugar consumption. This in turn promotes conversion to acids, which could improve the suitability of the model to describe the experimental data. In the latter cases (high initial glucose concentrations) the model shows a very marked dependence toward some parameters: *a*_1_, *v*_*P*3_, *n*_*AA*_, and *ATP*_*mid*_. As regards *a*_1_, its considerable sensitivity arises from the nature of the response curve to which it is linked. Indeed, *a*_1_ is the minimum value at which the logistic function tends in the presence of high concentrations of *G*, therefore, increasing the parameter means canceling the uptake while decreasing it means speeding it up. The variables *v*_*P*3_ and *n*_*AA*_ are related to the maximum rate of secondary acid production and to the amount of *ATP* consumed for the extrusion of AA. Finally, *ATP*_*mid*_ represents the concentration of *ATP* at which the logistics of the energy response curve assumes a value of 0.5. Clearly it is an extremely sensitive parameter since it intervenes in a crucial way on the beginning of the energy crisis.

## 4 Discussion

Recently, a report was published by Nova Institut, which highlighted the key drivers and inhibitors for bio-based *SA* production growth and market success (Chinthapalli et al., 2019). The industrial development of bio-based *SA* production processes mainly depends on the following key actions: (i) the selection of a proper *SA*-producing microorganism (Choi et al., 2016); (ii) the selection of a cost-effective and abundant biomass to produce hexose and pentose sugars *via* thermochemical or enzymatic treatments (Stylianou et al., 2020; Ferone et al., 2019a); (iii) the development of a reliable bioreactor characterized by high throughput (Ferone et al., 2019b); (iv) the determination of optimal operating conditions for *SA* production (Meynial-Salles et al., 2008); (v) the development of an effective recovery process (De Wever and Dennewald, 2018; Salma et al., 2021). As regards the key issue iv, kinetic modeling is considered a necessary step in developing a fermentation process since the models can be used to determine optimal operating conditions to produce a target metabolite.

In our view, system dynamics modeling approach represents a relevant tool to understand interactions underlying metabolic processes. In previous modeling works on yeast and bacteria model organisms, we demonstrated how a very simplified process-based model could be used to represent with high robustness microbial growth, metabolic shift between fermentation and respiration, and the onset of inhibition in high cell density cultures due to the secretion and accumulation of an inhibitory compound in the culture medium (Carteni et al., 2020; Mazzoleni et al., 2015).

In this work, the presented process-based model is able to represent the complex interactions between the starting concentration levels of the carbon source (glucose) in the growth medium and the dynamic metabolic status of the cells. In the model a pivotal role for *ATP*/*ADP* balance inside the cell is assumed. The rationale behind this is that the acidity of the fermentation products accumulating during the process, affects the energy costs of the cell, due to the energy demand for the secretion of acids and the maintenance of the intracellular pH (Russell and Cook, 1995; van Bodegom, 2007). Thus, as glucose concentration increases, acid production increases, resulting in a higher ATP consumption up to provoking an energy crisis inside the cell. This critical condition determines the slowing down of the physiological processes leading to growth so that less microbial mass is produced. However, glycolysis (*Gly1*) is still active and continues to deplete intracellular ATP. Finally, to avoid a complete energy crisis leading to cell death, the uptake of glucose is stopped, so that glucose remains unconsumed in the culture medium. The model takes into account this behavior, assuming that *A. succinogenes* is able to finely regulate the uptake of glucose. Such regulation is typical of the so-called Crabtree negative species (Shimizu and Matsuoka, 2019), which differ from the Crabtree positive ones, such as the yeast *S. cerevisiae*, in which the necessity to avoid sugar-induced cell death is addressed shifting metabolism from respiration to fermentation (de Alteriis et al., 2018).

On the basis of such assumptions, the proposed model well describes the observed experimental behavior of *A. succinogenes* in batch cultures as a function of initial glucose concentration, showing that a maximum of succinic acid is obtained between 50 and 60 g L^−1^ of initial glucose (Figure 5). This optimal initial glucose concentration matches the experimental evidence by other authors in batch cultures (Liu et al., 2008). The maintenance of residual glucose below a critical concentration, as the model suggests, can be easily achieved in a fed-batch reactor, by using a controlled glucose feeding. Indeed, this operative strategy has been reported to enhance SA concentration and productivity (Liu et al., 2008; Ferone et al., 2019a).

However, some significant deviations between simulated and measured acids productions were observed in specific culture conditions. In particular, regarding AA and FA the higher discrepancy between simulations and observations happens at the lowest glucose concentration conditions (G1) where all the productions are underestimated by the model. Regarding SA, the highest discrepancy is observed in the initial phases of the G2 experiment, although the final production of succinic acid is correctly simulated (Figure 3). This likely reflects the lack of an explicit NADH/NAD^+^ balance and its role in coupling the SA production (C4 pathway) with the FA and AA pathways (C3 pathway). The C3 pathway with formation of acetate and formate is indeed necessary for the production of the additional reducing power (NADH) needed for SA formation by the C4 pathway. In conclusion, the coupling between these different pathways through the NADH/NAD^+^ balance might further improve the dynamics of acids productions and, in particular, reduce the early overproduction of SA estimated by the model simulations.

## 5 Conclusions

The presented model was able to effectively reproduce the production of succinic acid and the corresponding dynamic trends of microbial growth and glucose consumption. In particular, it shows that the maximal production of succinic acid is achieved in conditions leading to glucose uptake limitation. The mechanism of metabolic regulation is assumed to be mainly associated with the ATP/ADP balance, dynamically explaining the effect of excess glucose on microbial growth and production. Further refinement of the model should also explicitly consider the role of NADH/NAD^+^ balance in the coupling of the metabolic pathways leading to acids production.

Additional investigation is necessary to validate the presented modeling assumptions, specifically the effect of the energy balance on metabolic regulation, by measuring the concentration of intracellular ATP and ADP in different stages of the population growth cycle.

In conclusion, the process-based modeling approach has revealed a strategic tool to effectively describe the main metabolic processes and their regulation, and consequently useful to better define working conditions and overcome the criticalities of the SA fermentation process.

## Data Availability

The datasets analyzed for this study can be found in Ferone et al. (2017): https://doi.org/10.1007/s12010-017-2514-4.

## References

[B1] Akhtar J. Idris A. Abd. Aziz R. (2014). Recent advances in production of succinic acid from lignocellulosic biomass. Appl. Microbiol. Biotechnol. 98, 987–1000. 10.1007/s00253-013-5319-6 24292125

[B2] Almqvist H. Pateraki C. Alexandri M. Koutinas A. Lidén G. (2016). Succinic acid production by *Actinobacillus succinogenes* from batch fermentation of mixed sugars. J. Indus. Microbiol. Biotechnol. 43, 1117–1130. 10.1007/s10295-016-1787-x 27255975

[B3] Boiteux A. Hess B. (1981). Design of glycolysis. Philos. Transac. Royal Soc. Lond. B Biol. Sci. 293, 5–22. 10.1098/rstb.1981.0056 6115423

[B4] Bradfield M. F. A. Nicol W. (2014). Continuous succinic acid production by *Actinobacillus succinogenes* in a biofilm reactor: steady-state metabolic flux variation. Biochem. Eng. J. 85, 1–7. 10.1016/j.bej.2014.01.009

[B5] Carteni F. Occhicone A. Giannino F. Vincenot C. E. de Alteriis E. Palomba E.. (2020). A general process-based model for describing the metabolic shift in microbial cell cultures. Front. Microbiol. 11:521368. 10.3389/fmicb.2020.521368 33117301 PMC7561435

[B6] Chatterjee R. Millard C. S. Champion K. Clark D. P. Donnelly M. I. (2001). Mutation of the *ptsG* Gene Results in Increased Production of Succinate in Fermentation of Glucose by *Escherichia coli*. Appl. Environ. Microbiol. 67, 148–154. 10.1128/AEM.67.1.148-154.2001 11133439 PMC92534

[B7] Chinthapalli R. Puente Á. Skoczinski P. Raschka A. Carus M. (2019). Succinic Acid: From a Promising Building Block to a Slow Seller—What Will a Realistic Future Market Look Like? Available online at: http://www.bio-based.eu/reports/ (accessed October 15 2024).

[B8] Choi S. Song H. Lim S. W. Kim T. Y. Ahn J. H. Lee J. W.. (2016). Highly selective production of succinic acid by metabolically engineered *Mannheimia succiniciproducens* and its efficient purification. Biotechnol. Bioeng. 113, 2168–2177. 10.1002/bit.25988 27070659

[B9] Corona-González R. I. Bories A. González-Álvarez V. Pelayo-Ortiz C. (2008). Kinetic study of succinic acid production by *Actinobacillus succinogenes* ZT-130. Process Biochem. 43, 1047–1053. 10.1016/j.procbio.2008.05.011 19777303

[B10] de Alteriis E. Cartenì F. Parascandola P. Serpa J. Mazzoleni S. (2018). Revisiting the Crabtree/Warburg effect in a dynamic perspective: a fitness advantage against sugar-induced cell death. Cell Cycle, 17, 688–701. 10.1080/15384101.2018.1442622 29509056 PMC5969562

[B11] de Alteriis E. Incerti G. Cartenì F. Chiusano M. L. Colantuono C. Palomba E.. (2023). Extracellular DNA secreted in yeast cultures is metabolism-specific and inhibits cell proliferation. Microbial Cell. 10, 277–295. 10.15698/mic2023.12.810 38053574 PMC10695634

[B12] De Deken R. H. (1966). The crabtree effect: a regulatory system in yeast. J. Gen. Microbiol. 44, 149–156. 10.1099/00221287-44-2-149 5969497

[B13] de Falco B. Giannino F. Carteni F. Mazzoleni S. Kim D.-H. (2022). Metabolic flux analysis: a comprehensive review on sample preparation, analytical techniques, data analysis, computational modelling, and main application areas. RSC Adv. 12, 25528–25548. 10.1039/D2RA03326G 36199351 PMC9449821

[B14] De Wever H. Dennewald D. (2018). Screening of sorbents for recovery of succinic and itaconic acid from fermentation broths. J. Chem. Technol. Biotechnol. 93, 385–391. 10.1002/jctb.5366

[B15] Ercole A. Raganati F. Salatino P. Marzocchella A. (2021). Continuous succinic acid production by immobilized cells of *Actinobacillus succinogenes* in a fluidized bed reactor: entrapment in alginate beads. Biochem. Eng. J. 169:107968. 10.1016/j.bej.2021.107968

[B16] Ferone M. Ercole A. Raganati F. Olivieri G. Salatino P. Marzocchella A.. (2019a). Efficient succinic acid production from high-sugar-content beverages by *Actinobacillus succinogenes*. Biotechnol. Prog. 35:e2863. 10.1002/btpr.2863 31173476

[B17] Ferone M. Raganati F. Olivieri G. Marzocchella A. (2019b). Bioreactors for succinic acid production processes. Crit. Rev. Biotechnol. 39, 571–586. 10.1080/07388551.2019.1592105 30931643

[B18] Ferone M. Raganati F. Olivieri G. Salatino P. Marzocchella A. (2017). Biosuccinic acid from lignocellulosic-based hexoses and pentoses by *Actinobacillus succinogenes*: characterization of the conversion process. Appl. Biochem. Biotechnol. 183, 1465–1477. 10.1007/s12010-017-2514-4 28540516

[B19] Forrester J. (1961). Industrial Dynamics. Encino: Pegasus Communications.

[B20] Fukuzaki S. Nishio N. Shobayashi M. Nagai S. (1990). Inhibition of the fermentation of propionate to methane by hydrogen, acetate, and propionate. Appl. Environ. Microbiol. 56, 719–723. 10.1128/aem.56.3.719-723.1990 16348146 PMC183412

[B21] Lagarias J. C. Reeds J. A. Wright M. H. Wright P. E. (1998). Convergence properties of the nelder–mead simplex method in low dimensions. SIAM J. Optimiz. 9, 112–147. 10.1137/S1052623496303470

[B22] Li Q. Wang D. Wu Y. Yang M. Li W. Xing J.. (2010). Kinetic evaluation of products inhibition to succinic acid producers *Escherichia coli* NZN111, AFP111, BL21, and *Actinobacillus succinogenes* 130ZT. J. Microbiol. 48, 290–296. 10.1007/s12275-010-9262-2 20571945

[B23] Lin S. K. C. Du C. Koutinas A. Wang R. Webb C. (2008). Substrate and product inhibition kinetics in succinic acid production by *Actinobacillus succinogenes*. Biochem. Eng. J. 41, 128–135. 10.1016/j.bej.2008.03.013

[B24] Liu Y. Zheng P. Sun Z. Ni Y. Dong J. Wei P.. (2008). Strategies of pH control and glucose-fed batch fermentation for production of succinic acid by *Actinobacillus succinogenes* CGMCC1593. J. Chem. Technol. Biotechnol. 83, 722–729. 10.1002/jctb.1862

[B25] Mazzoleni S. Landi C. Cartenì F. de Alteriis E. Giannino F. Paciello L.. (2015). A novel process-based model of microbial growth: self-inhibition in *Saccharomyces cerevisiae* aerobic fed-batch cultures. Microb. Cell Fact. 14:109. 10.1186/s12934-015-0295-4 26223307 PMC4518646

[B26] McKinlay J. B. Shachar-Hill Y. Zeikus J. G. Vieille C. (2007). Determining *Actinobacillus succinogenes* metabolic pathways and fluxes by NMR and GC-MS analyses of 13C-labeled metabolic product isotopomers. Metab. Eng. 9, 177–192. 10.1016/j.ymben.2006.10.006 17197218

[B27] Meynial-Salles I. Dorotyn S. Soucaille P. (2008). A new process for the continuous production of succinic acid from glucose at high yield, titer, and productivity. Biotechnol. Bioeng. 99, 129–135. 10.1002/bit.21521 17546688

[B28] Mor I. Cheung E. C. Vousden K. H. (2011). Control of glycolysis through regulation of PFK1: old friends and recent additions. Cold Spring Harb. Symp. Quant. Biol. 76, 211–216. 10.1101/sqb.2011.76.010868 22096029

[B29] Morris M. D. (1991). Factorial sampling plans for preliminary computational experiments. Technometrics. 33, 161–174. 10.1080/00401706.1991.10484804

[B30] Neumann S. Grosse K. Sourjik V. (2012). Chemotactic signaling *via* carbohydrate phosphotransferase systems in *Escherichia coli*. Proc. Nat. Acad. Sci. 109, 12159–12164. 10.1073/pnas.1205307109 22778402 PMC3409764

[B31] Norton J. (2015). An introduction to sensitivity assessment of simulation models. Environ. Modell. Softw. 69, 166–174. 10.1016/j.envsoft.2015.03.020

[B32] Paczia N. Nilgen A. Lehmann T. Gätgens J. Wiechert W. Noack S.. (2012). Extensive exometabolome analysis reveals extended overflow metabolism in various microorganisms. Microb. Cell Fact. 11:122. 10.1186/1475-2859-11-122 22963408 PMC3526501

[B33] Pateraki C. Almqvist H. Ladakis D. Lidén G. Koutinas A. A. Vlysidis A.. (2016a). Modelling succinic acid fermentation using a xylose based substrate. Biochem. Eng. J. 114, 26–41. 10.1016/j.bej.2016.06.01137395870

[B34] Pateraki C. Patsalou M. Vlysidis A. Kopsahelis N. Webb C. Koutinas A. A.. (2016b). *Actinobacillus succinogenes*: advances on succinic acid production and prospects for development of integrated biorefineries. Biochem. Eng. J. 112, 285–303. 10.1016/j.bej.2016.04.005

[B35] Plumbridge J. (1998). Expression of *ptsG*, the gene for the major glucose PTS transporter in *Escherichia coli*, is repressed by Mlc and induced by growth on glucose. Mol. Microbiol. 29, 1053–1063. 10.1046/j.1365-2958.1998.00991.x 9767573

[B36] Prabhu A. A. Ledesma-Amaro R. Lin C. S. K. Coulon F. Thakur V. K. Kumar V.. (2020). Bioproduction of succinic acid from xylose by engineered Yarrowia lipolytica without pH control. Biotechnol. Biofuels. 13:113. 10.1186/s13068-020-01747-3 32607128 PMC7321536

[B37] Rigaki A. Webb C. Theodoropoulos C. (2020). Double substrate limitation model for the bio-based production of succinic acid from glycerol. Biochem. Eng. J. 153:107391. 10.1016/j.bej.2019.107391

[B38] Russell J. B. Cook G. M. (1995). Energetics of bacterial growth: balance of anabolic and catabolic reactions. Microbiol. Rev. 59, 48–62. 10.1128/mr.59.1.48-62.1995 7708012 PMC239354

[B39] Salma A. Djelal H. Abdallah R. Fourcade F. Amrane A. (2021). Platform molecule from sustainable raw materials; case study succinic acid. Braz. J. Chem. Eng. 38, 215–239. 10.1007/s43153-021-00103-8

[B40] Samuoelov N. S. Datta R. Jain M. K. Zeikus J. (1990). Microbial decarboxylation of succinate to propionate. Ann. N. Y. Acad. Sci. 589, 697–704. 10.1111/j.1749-6632.1990.tb24282.x

[B41] Sánchez A. M. Bennett G. N. San K-. Y. (2005). Novel pathway engineering design of the anaerobic central metabolic pathway in *Escherichia coli* to increase succinate yield and productivity. Metab. Eng. 7, 229–239. 10.1016/j.ymben.2005.03.001 15885621

[B42] Shimizu K. Matsuoka Y. (2019). Regulation of glycolytic flux and overflow metabolism depending on the source of energy generation for energy demand. Biotechnol. Adv. 37, 284–305. 10.1016/j.biotechadv.2018.12.007 30576718

[B43] Song H. Jang S. H. Park J. M. Lee S. Y. (2008). Modeling of batch fermentation kinetics for succinic acid production by *Mannheimia succiniciproducens*. Biochem. Eng. J. 40, 107–115. 10.1016/j.bej.2007.11.021

[B44] Song H. Lee S. Y. (2006). Production of succinic acid by bacterial fermentation. Enzyme Microb. Technol. 39, 352–361. 10.1016/j.enzmictec.2005.11.043

[B45] Stylianou E. Pateraki C. Ladakis D. Cruz-Fernández M. Latorre-Sánchez M. Coll C.. (2020). Evaluation of organic fractions of municipal solid waste as renewable feedstock for succinic acid production. Biotechnol. Biofuels. 13:72. 10.1186/s13068-020-01708-w 32322302 PMC7160979

[B46] van Bodegom P. (2007). Microbial maintenance: a critical review on its quantification. Microb. Ecol. 53, 513–523. 10.1007/s00248-006-9049-5 17333428 PMC1915598

[B47] Vemuri G. N. Eiteman M. A. Altman E. (2002). Effects of growth mode and pyruvate carboxylase on succinic acid production by metabolically engineered strains of *Escherichia coli*. Appl. Environ. Microbiol. 68, 1715–1727. 10.1128/AEM.68.4.1715-1727.2002 11916689 PMC123851

[B48] Vlysidis A. Binns M. Webb C. Theodoropoulos C. (2011). Glycerol utilisation for the production of chemicals: Conversion to succinic acid, a combined experimental and computational study. Biochem. Eng. J. 58–59, 1–11. 10.1016/j.bej.2011.07.004

[B49] Wang J. Zhang R. Zhang Y. Yang Y. Lin Y. Yan Y.. (2019). Developing a pyruvate-driven metabolic scenario for growth-coupled microbial production. Metab. Eng. 55, 191–200. 10.1016/j.ymben.2019.07.011 31348998 PMC6744941

[B50] Wisselink H. W. Toirkens M. J. del Rosario Franco Berriel M. Winkler A. A. van Dijken J. P. Pronk J. T.. (2007). Engineering of *Saccharomyces cerevisiae* for efficient anaerobic alcoholic fermentation of L-Arabinose. Appl. Environ. Microbiol. 73, 4881–4891. 10.1128/AEM.00177-07 17545317 PMC1951023

[B51] Wolfe A. J. (2005). The acetate switch. Microbiol. Mol. Biol. Rev. 69, 12–50. 10.1128/MMBR.69.1.12-50.2005 15755952 PMC1082793

[B52] Zhou J. Liu L. Shi Z. Du G. Chen J. (2009). ATP in current biotechnology: regulation, applications and perspectives. Biotechnol. Adv. 27, 94–101. 10.1016/j.biotechadv.2008.10.005 19026736

[B53] Zhu X. Tan Z. Xu H. Chen J. Tang J. Zhang X.. (2014). Metabolic evolution of two reducing equivalent-conserving pathways for high-yield succinate production in *Escherichia coli*. Metab. Eng. 24, 87–96. 10.1016/j.ymben.2014.05.003 24831708

